# *CSD Communications* of the Cambridge Structural Database

**DOI:** 10.1107/S2052252522010545

**Published:** 2023-01-01

**Authors:** Gregory M. Ferrence, Clare A. Tovee, Stephen J.W. Holgate, Natalie T. Johnson, Matthew P. Lightfoot, Kamila L. Nowakowska-Orzechowska, Suzanna C. Ward

**Affiliations:** a Department of Chemistry, Illinois State University, Normal, IL 61790-4160, USA; b Cambridge Crystallographic Data Centre, 12 Union Road, Cambridge CB2 1EZ, United Kingdom; University of St Andrews, United Kingdom

**Keywords:** Cambridge Structural Database, *CSD Communications*, data preservation

## Abstract

The Cambridge Structural Database (CSD) is a collection of over one million experimental three-dimensional structures obtained through crystallographic analyses. These structures are determined by crystallographers worldwide and undergo curation and enhancement by scientists at the Cambridge Crystallographic Data Centre (CCDC) prior to their addition to the database. Though the CSD is substantial and contains widespread chemical diversity across organic and metal–organic compounds, it is estimated that a significant proportion of crystal structures determined are not published or shared through the peer-reviewed journal mechanism. To help overcome this, scientists can publish structures directly through the database as *CSD Communications* and these structural datasets are made publicly available alongside structures associated with scientific articles. *CSD Communications* contribute to the collective crystallographic knowledge as nearly two thirds are novel structures that are not otherwise available in the scientific literature. The primary benefits of sharing data through *CSD Communications* include the long-term preservation of scientific data, the strengthening of a widely data-mined world repository (the CSD), and the opportunity for scientists to receive recognition for their work through a formal and citable data publication. All *CSD Communications* are assigned unique digital object identifiers (DOIs). Contributions as *CSD Communications* currently comprise about 3.89% of the total CSD entries. Each individual *CSD Communication* is free to view and retrieve from the CCDC website.

## Abstract 2

*CSD Communications* have opened up a new pathway for sharing crystallographic data that are unlikely to be published in a scientific article, contributing to both the breadth and the depth of the CSD. This review discusses the benefits, challenges, mechanism and trends in these structures.

## 1. Introduction

*CSD Communications* provide opportunities to share crystal structures with the scientific community directly through the Cambridge Structural Database (CSD) without an accompanying article in the scientific literature (CCDC, 2022*a* ▸). Relative to the large number of possible structures in ‘chemical space’, crystallographic databases have substantial gaps in coverage. Simultaneously there exists a longstanding mindset that striving to lessen such gaps is meritorious (Strasser, 2019 ▸; Helliwell, 2022 ▸). Two decades ago, conjecture within the crystallographic community suggested that plausibly as few as 20% of publishable crystal structures had been published (Coles *et al.*, 2005 ▸). For over forty years, the number of entries in the CSD has approximately doubled each decade to nearly 1.2 million at present (Allen, 2002 ▸; Groom *et al.*, 2016 ▸). With ever-increasing access to better, faster and more crystallography instrumentation, the challenge of publishing structures at a rate comparable to high-throughput crystallographic data collection continues to escalate (Allen, 2004 ▸). A fact in stark contrast to the ideal expressed by Coles *et al.* (2020 ▸): ‘if a compound has been synthesized and there is ready access to crystallography, then why should not its crystal structure be determined in order to contribute to the body of knowledge?’ In a recent *IUCr Newsletter* (Clegg, 2020 ▸), the author concurs that only a minority of structures solved are in databases and details eleven compelling reasons why structures are not published. Publishing crystallographic data as a *CSD Communication* has clear benefits in giving recognition to the authors for the work and complying with open access mandates from some funders. There have been other efforts to address the growing number of crystal structures from a variety of publishers. These include *Zeitschrift für Kristallographie – New Crystal Structures* from De Gruyter, *Crystals* from MDPI and several IUCr journals, particularly *Acta E* and *IUCrData* which are devoted to publishing crystal structures without the need to report other spectroscopic analyses (Harrison *et al.*, 2016 ▸). *CSD Communications* also fit alongside similar initiatives by other crystallographic databases including *ICSD Communications* which are found in the Inorganic Crystal Structure Database (ICSD; Bergerhoff & Brown, 1987 ▸).

### 1.1. Evolution of *CSD Communications*

The first structures to be included in the CSD without an accompanying article were originally known as ‘private communications’. The earliest private communication, by publication year, dates to 1976 (Woolf, 1976 ▸) and by 1990 only 13 CSD entries were listed as private communications out of a total 104 328 in the database. There was a slow but steady increase over the following decades and by 2016 the collection had grown to over 15 000 entries. At this time the decision was taken to rename these as *CSD Communications* to better reflect that these are public not private datasets which are included in the CSD for the benefit of the crystallographic community.

**Figure 1:**
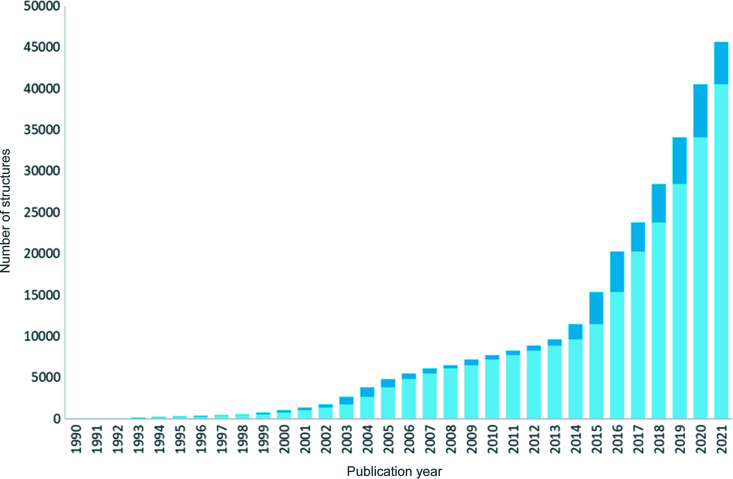
Chart showing the growth in the number of *CSD Communications*. The stacked bars represent the number of structures added that year in dark blue and the cumulative number of structures from previous years in light blue.

Since then, growth of the collection has accelerated (Fig. 1 ▸). In the latest release of the CSD (version 5.43 plus March and June 2022 updates), of a total 1 197 342 database entries, 46 616 are *CSD Communications*. Between 2019–2021 over 5000 new structures were published each year as *CSD Communications*.

Efforts to advertise *CSD Communications* in the crystallographic community, the engagement of several prolific crystallographers and growth in correspondence encouraging release of embargos on deposited structures have contributed to the increased number of *CSD Communications* now shared. During the last decade, to support crystallographers in sharing more data, the CCDC also implemented a CIF deposition and validation web-portal. Together these efforts have contributed to *CSD Communications* becoming the number one platform to publish crystallographic data since 2018.

To aid the discoverability and accessibility of published structures archived in the CSD, the CCDC began assigning datasets a unique digital object identifier (DOI) in 2014. Although publications reporting multiple crystal structures are typically associated with an article DOI for the entire publication, each individual crystal structure in a publication is assigned its own unique dataset DOI. This provides individual structures in the CSD with a permanent access link which can be easily referenced along with the CCDC number in a publication. For data depositors, this provides more recognition and visibility for their *CSD Communications.* For authors, dataset DOIs allow referencing of a specific crystal structure from a larger published work and, in the case of *CSD Communications*, provide a clear mechanism for citation in their own publications. In response to requests by crystallographers and to provide publishers and academic institutions with a convenient mechanism for tracking and recording *CSD Communications*, in 2019 an electronic International Standard Serial Number (ISSN 2631–9888) was assigned to *CSD Communications*. Additionally, the collection of *CSD Communications* is available via a journal-style archive of structures by year at https://www.ccdc.cam.ac.uk/Community/csd-communications/.

### 1.2. Author and community benefits

There are a variety of reasons that authors may decide to publish *CSD Communications*, including:

(1) There is no other forthcoming publication incorporating the crystallographic data planned.

(2) The crystal structure has become orphaned and is stand-alone, perhaps due to prior publication of the relevant chemistry.

(3) To share re-determined data where the chemical compound is already in the CSD, but the dataset is still of value for comparative studies, for example it was collected under different conditions, or it is of higher quality.

(4) A crystallographer chooses to disseminate unpublished data on retirement.

(5) A crystallographer sharing data collected for a collaborator and/or principal investigator who has released the data for dissemination since there is no expectation of publication elsewhere.

There are many benefits to sharing data through *CSD Communications*. It provides crystallographers and authors with the opportunity to receive credit for their work through a citable and persistent publication mechanism. The publication of *CSD Communications* also ensures long-term data preservation according to accepted international standards, namely in the manner conforming with the rules of the CCDC Data Preservation Policy (CCDC, 2022*b* ▸). Some funding agencies such as Research Councils in the UK require data produced for grants to be shared in an appropriate repository (UKRI, https://www.ukri.org/wp-content/uploads/2020/10/UKRI-020920-OpenAccessPolicy.pdf). The CCDC is certified by CoreTrustSeal (CTS, 2020 ▸) and is a recognized repository of crystallographic data providing the citable references for their users including the data DOI and the ISSN (2631-9888) for the *CSD Communication* data collection (https://portal.issn.org/resource/ISSN/2631-9888).

**Figure 2:**
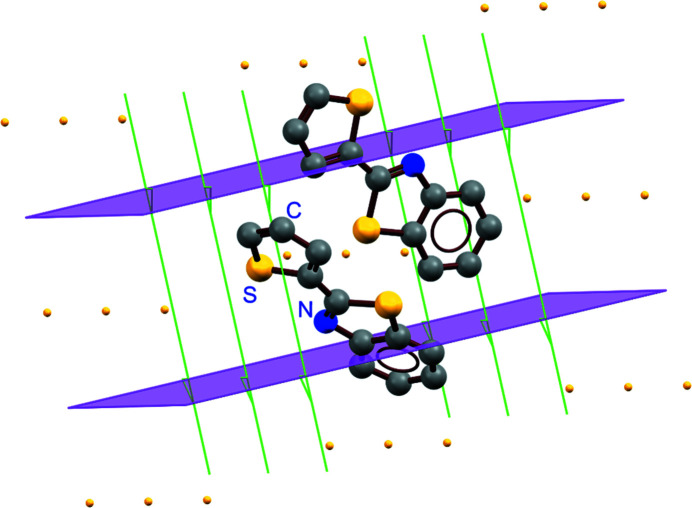
*Mercury* (Macrae *et al.*, 2020 ▸) rendering highlighting space-group symmetry elements of the *Z*′ = 2 polymorph CSD-CAMBAV.

Depositing and sharing data as a *CSD Communication* allows other scientists to benefit from these structures and gain insights from the reuse of data. For example, a paper (Clegg, 2019 ▸) discussing polymorphs of thio­phene-substituted benzo­thia­zoles includes a *CSD Communication*, CSD-CAMBAV (Renz *et al.*, 2011 ▸). Without this structure (Fig. 2 ▸), the record of known polymorphs would be incomplete and could change the conclusion of research from data mining. This is not an isolated example; citations to *CSD Communications* can be found in many journal articles in the chemistry literature.

This review highlights some aspects and recent developments associated with the *CSD Communication* mechanism, with a view to increasing the uptake of *CSD Communications* as a means of sharing crystallographic data within the wider crystallographic and scientific communities.

## 2. What structures are *CSD Communications*?

The CSD is a compendium of essentially all published small-molecule-organic and metal–organic crystal structures, where metal–organic indicates that the structure contains a metal and an organic ligand, molecule or ion. This section aims to demonstrate the extremely diverse types of structures that are shared as *CSD Communications*, compare these with the CSD and discuss some of the trends observed over time.

### 2.1. Trends within CSD Communications

#### 2.1.1. What type of chemical structures are *CSD Communications*?

**Figure 3:**
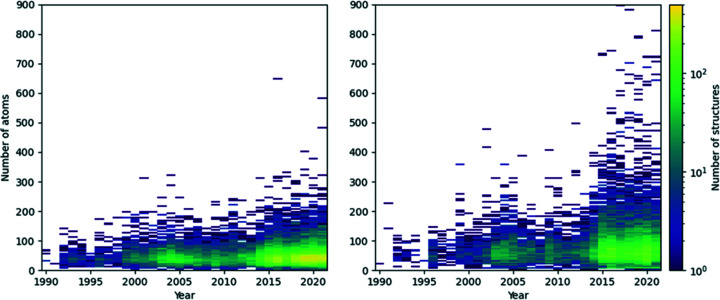
Frequency heat maps of the number of atoms per structure over time for organic (left) and metal–organic (right) crystal structures.

It is not just the number of structures that has been increasing over time, their size and complexity have also increased, especially in the last 5 years. Starting with a broad look at the proportion of *CSD Communications* structures that are organic or metal–organic, 57% of *CSD Communications* structures are organic compared with only 44% of structures in the whole CSD. The balance has fluctuated each year since 1990, as shown in Fig. S1 of the supporting information. The plots in Fig. 3 ▸ show how the number of atoms per structure, in the largest chemical unit, has increased over the last 30 years. In the left-hand plot the number of organic *CSD Communications* increases over time as illustrated by the bright yellow section and a slight increase in the range of the number of atoms per structure as shown in the bars higher on the *y* axis is provided. A similar trend is observed for metal–organic structures in the right-hand plot, although there is a greater variation in the size of the structures, as might be expected for metal–organic entries. Some of these metal–organic structures have over 800 atoms, *i.e.* CSD-HISSOU (Yuan, 2018 ▸).

When the chemical nature of *CSD Communications* are considered, there are 44 678 unique chemical structures (as indicated by the number of different CSD refcode families) and the structures contain 84 different elements. There are 10 elements that appear in the CSD but not in any *CSD Communications* (He, Ne, Ar, Xe, Pm, Ac, Pa, Cm, Bk and Cf). This is perhaps not surprising since cumulatively they represent <0.02% of the structures in the CSD and therefore any new structure containing one of these elements is likely to warrant a description in an associated scientific article. *CSD Communications* also feature in a list of structures in the CSD with the most different elements in an individual structure, with 19 structures containing 10 different elements each, including the rhodium complex CSD-JOKSAG by Rheingold (2019 ▸). This feat is only 2 elements fewer than the current record in the CSD of 12 unique elements in a single structure, which occurs in entries CSD-CIGTOC (Boyer *et al.*, 2007 ▸), CSD-KEDMEP and CSD-KEDMOZ (Ng *et al.*, 2022 ▸).

**Figure 4:**
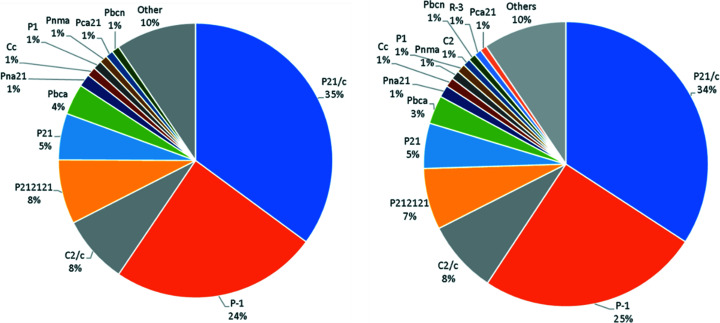
Pie charts showing the proportions of space groups for *CSD Communications* (left) and the CSD (right).

Mining the data also shows that *CSD Communications* are found to be in nearly 200 different space groups with the most frequent space group observed as *P*2_1_/*c*. The top 5 space groups are consistent and are in similar proportions between *CSD Communications* and the CSD; a comparison is illustrated in the pie charts in Fig. 4 ▸. Experimental techniques are well covered in *CSD Communications*, with 651 structures identified as having been measured with synchrotron radiation, 5 neutron radiation studies and 58 structures determined at high pressure. The majority of *CSD Communications* structures were determined at low temperatures.

#### 2.1.2. Diversity of structures in *CSD Communications*

Nearly two thirds of *CSD Communications* contain chemical substances that cannot be found outside of the *CSD Communications* collection, as indicated by the percentage of refcode families (Groom *et al.*, 2016 ▸) that only contain *CSD Communications* (see Section S2 of the supporting information). In these cases, the structures can either contain completely novel molecules or be multi-component structures of a molecule already observed in another entry. Therefore, *CSD Communications* provide the CSD with many new and unique structures as well as new combinations of multi-component systems which may be of interest beyond the identification of the main molecule.

**Figure 5:**
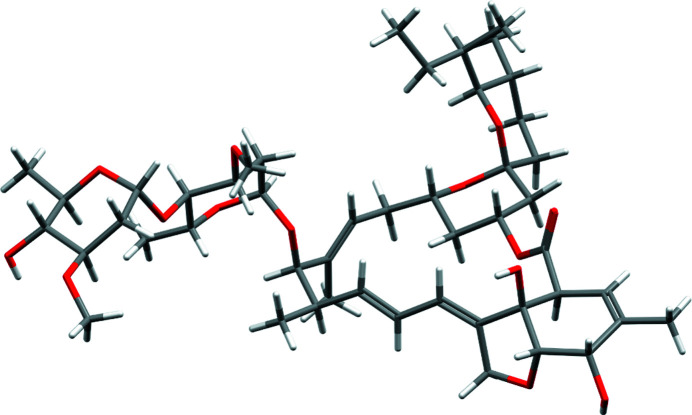
CSD ‘stick style’ *Mercury* rendering of CSD-BIFYOF.

*CSD Communications* cover a diverse range of chemistry as indicated by their inclusion in CSD subsets. Lists of CSD entries which focus on particular information or types of chemistry are aggregated to form CSD subsets. These lists help researchers to focus on areas of interest such as metal–organic frameworks (MOFs) (Moghadam *et al.*, 2017 ▸), pharmaceuticals (Bryant *et al.*, 2019 ▸), pesticides and compounds which have been investigated as targets against COVID-19. *CSD Communications* feature in all current CSD subsets, including the CSD-COVID19 subset, which contains 8 *CSD Communications* including CSD-ESOURE12 [estradiol, a steroid (Chen, 2018 ▸)] and CSD-BIFYOF [the primary avermectin in the antiparasitic agent ivermectin (Seppala *et al.*, 2005 ▸)], see Fig. 5 ▸. In general, the percentage of *CSD Communications* in the various CSD subsets is broadly similar to the percentage of the whole CSD in these subsets (see Table S1 of the supporting information). For example, the proportion of *CSD Communications* in the ‘best representative’ lists (van de Streek, 2006 ▸) are slightly higher than the CSD as a whole. However, the percentage of *CSD Communications* that are in the two MOF subsets is lower than that of the whole CSD.

#### 2.1.3. Data quality of *CSD Communications*

Crystal structures are limited in value if the data are of poor quality and therefore not a reliable basis for further research. Here, the *R* factor is considered, which is often quoted in any publication alongside crystal structure data. The average mean *R* factor for all *CSD Communications* is 5.05% compared with an average over the whole CSD of 5.13%, which is reassuring for the reliability and reuse of these structures. Further comparison of the variability by year is illustrated in Fig. S2. Previous work has compared *CSD Communications* with selected journals from relevant categories in Clarivate *Web of Science* (Tovee *et al.*, 2018 ▸). This study showed that they are similar in terms of the widely quoted *R* factor, the number of alerts generated by the IUCr checkCIF service (Spek, 2020 ▸) and the percentage of bonds or angles classified as unusual by the CCDC program *Mogul* (Bruno *et al.*, 2004 ▸).

## 3. How are *CSD Communications* added to the CSD?

### 3.1. What is included in a deposition?

#### 3.1.1. Depositing *CSD Communications*

The process for depositing a *CSD Communication* is very similar to the general author-/crystallographer-initiated deposition of a structure intended to be shared through an associated scientific article. The primary difference being to flag the deposition for sharing immediately through the CSD. The multistep online deposition process is described elsewhere (CCDC, 2022*c* ▸,*d* ▸) so this section will focus on the key differences for *CSD Communications*.

**Figure 6:**
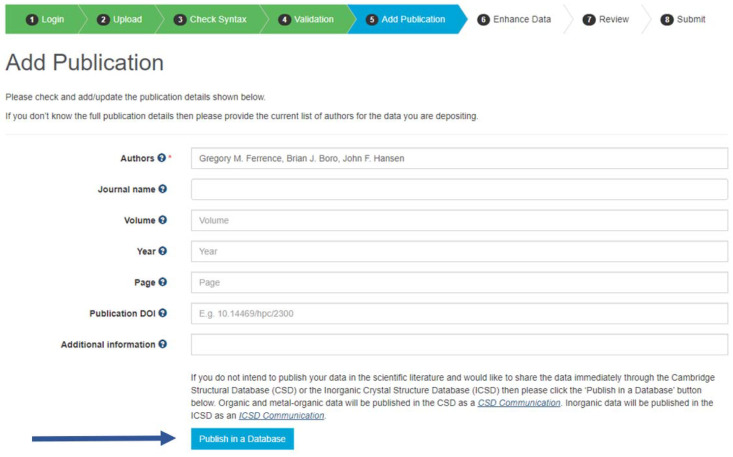
Specifying ‘Publish in a Database’ in the ‘Add Publication’ step of the CSD entry deposition process.

For *CSD Communications* the deposition process differs at the ‘Add Publication’ stage, where the depositor seeking to include the deposition for publication as a *CSD Communication* selects ‘Publish in a Database’ (Fig. 6 ▸). At this stage, accreditation of all researchers involved in the production of the crystal structure should also be recorded in the list of authors. This may include the crystallographer, as well as the principal investigator or supervisor, any additional data collection/data solution/refinement scientists, and experimentalists involved in the synthesis and recrystallization of the crystal.

With the structure flagged as a *CSD Communication*, the ‘Enhance Data’ stage provides an opportunity for the depositor to provide additional details. This stage is particularly valuable for *CSD Communications* since such details are unlikely to be disseminated elsewhere.

Once a structure is deposited as a *CSD Communication* and the deposition number has been assigned it will automatically be released to the public on the CCDC Access Structures website, possibly within a few minutes of submission (CCDC, 2022*e* ▸). This means that it is a fast method of publication.

**Figure 7:**
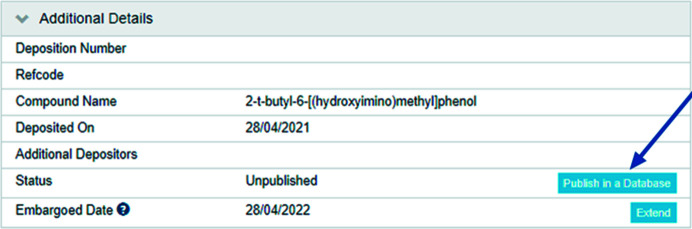
‘Publish in a Database’ for previously deposited data in ‘My Structures’.

In 2016 the CCDC ‘My Structures’ service was launched to enable depositors to view, retrieve, manage and share their deposited data. This service extended the functionality available to depositors and importantly added a mechanism for depositors to share previously deposited unpublished data as a *CSD Communication* (see Fig. 7 ▸). This means that crystallographers can now select unpublished data and share their work with the community more easily. Through this route, depositors still need to add all the authors who contributed to the structure. The publication year will be automatically set to the year that the data are shared.

#### 3.1.2. Validation and curation of *CSD Communications*

In general, the treatment of *CSD Communications* from deposition to validation and curation into the CSD is similar to structures published in associated journal articles but with a few differences, discussed here. For structures published as *CSD Communications*, the data are made publicly available immediately through the CCDC Access Structures service with a warning to users of the database that entries are undergoing validation.

Before inclusion in the desktop version of the CSD, every *CSD Communication* is checked for chemical and crystallographic sense by a member of the editorial team at the CCDC. This means that, since a *CSD Communication* does not have an accompanying journal article, the more information that can be provided during deposition, the better the finished database entry and the more searchable and usable the data will be. A crucial point in checking the chemical sense of the structure is charge balance. If there is any possibility for ambiguity, perhaps due to the chemistry being unusual, hydrogen atoms having not been located or for cases where metal oxidation states are open to interpretation, it is very useful for depositors to include more information about the chemical connectivity alongside the deposited dataset. It is also important to include information that may be hard to infer from the dataset. For example, if a solvent molecule has been treated using *SQUEEZE* (van der Sluis & Spek, 1990 ▸) or *MASK* (Jiang & Brünger, 1994 ▸), it is useful to include information about the species involved if they are known but it is almost equally valuable to know (actively) that the species were not identified. More information and guidelines regarding the deposition of structures as *CSD Communications* are available (CCDC, 2022*a* ▸). If there are any issues with the structure, the depositor may be contacted by the CCDC for further clarification.

### 3.2. How to cite *CSD Communications*

**Figure 8:**
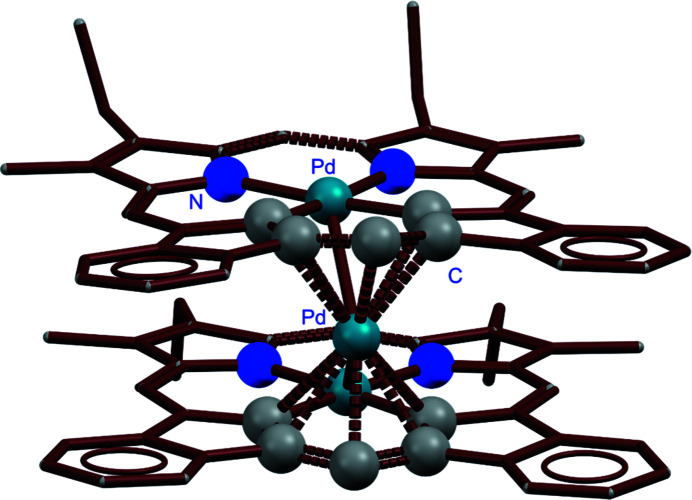
*Mercury* rendering highlighting the metal coordination environment of CSD-RIYGUD.

To cite structures from the CSD, it is recommended that the CSD refcode should be included within the body of the paper in the style CSD-REFCODE. For example, CSD-RIYGUD is an identifier for the crystal structure of an unusual ‘sandwich’ type palladium(0) metal complex (Fig. 8 ▸) (AbuSalim *et al.*, 2014 ▸). With the CSD identifier, a reader can locate this structure via a direct search of the CSD using the CCDC Access Structures service (CCDC, 2022*e* ▸), or using a resolution service such as http://identifiers.org/ (EMBL-EBI: European Molecular Biology Laboratory’s European Bioinformatics Institute) and inputting the identifier in the format CSD:RIYGUD. For structures published in a scientific article it is also recommended that the paper where the crystal structure is published is also cited (*i.e.* AbuSalim *et al.*, 2014 ▸). In the case of *CSD Communications*, the data DOI can be used in place of the article DOI, and ‘*CSD Communication*’ used in place of the journal name. For example,

Cati, D. S. & Stoeckli-Evans H. (2004). *CSD Communication*, CCDC 227635. https://doi.org/10.5517/cc7mw2s.

would be one reference format option for citing the asymmetrically coordinated di-copper complex [refcode CSD-ASEWEA (Cati & Stoeckli-Evans, 2004 ▸)]. The dataset may be cited directly by referring to the structure-specific data DOI (found on ‘Access Structures’), *i.e.*

AbuSalim, D. I., Ferrence, G. M. & Lash T. D. (2014). *Experimental Crystal Structure Determination*, CCDC 989317. https://doi.org/10.5517/cc126gg6.

The recommended format for references varies between publishers and journals. The CSD data citation format is similar to that recommended by DataCite. The only additional field that is included in the DataCite format is the publisher, in this case, Cambridge Crystallographic Data Centre (DataCite, https://datacite.org/cite-your-data.html).

## 4. Origins of *CSD Communications*

### 4.1. Where do *CSD Communications* come from?

**Figure 9:**
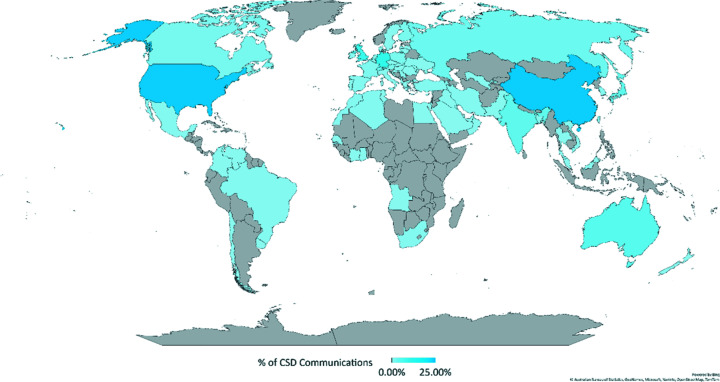
Location of authors of *CSD Communications*. Colour strength based on percentage of *CSD Communications* from the country.

Information about the crystallographer, including the country of residence, is required during web deposition. This information can be used to build a picture showing from which parts of the world *CSD Communications* are shared. The map in Fig. 9 ▸ highlights the countries and the relative number of *CSD Communication* depositions over the last 3 years. There are currently two leaders: China and the USA, totalling 48% of all the *CSD Communications* depositions; however, the number of unique depositors per country differs significantly between the two. Most datasets from USA come from a small percentage of all American users, whereas the Chinese *CSD Communications* originate from many more crystallographers. This is demonstrated by the fact that the top American depositor of *CSD Communications*, Professor Arnold Rheingold, with 2100 structures shared in this way, has the same number of *CSD Communications* as the top 10 depositors from China combined.

### 4.2. *CSD Communications* initiatives

Although the option to publish data directly through the CSD without an associated scientific article has been available for over 40 years, it is likely there are still depositors who remain unaware of this initiative. Many depositors discover the possibility via social media, conferences, discussions with colleagues or through annual emails sent by the CCDC about unpublished deposited datasets. These annual emails began in 2016 and each year the CCDC sends thousands of email reminders to depositors to inquire about the outcome of the unpublished datasets. These emails encourage depositors to share these data through the CSD as *CSD Communications*.

The emails were established when the scale of unpublished data in the CCDC internal repository became apparent following the introduction of a new internal system to manage data. In the first year of this scheme, depositors were contacted regarding over 66 000 structures. The emails resulted in the publication of an additional 4000 *CSD Communications* (alongside notifications about previously undiscovered associated scientific articles and requests to extend embargo periods), which formed the majority of the *CSD Communications* published in 2016. Since this time, the collection of unpublished datasets has reduced, but nevertheless, between 2017 and 2021, over 1500 crystal structures were published as *CSD Communications* as a direct result of these emails. This is still a sizeable number of structures that would have otherwise remained unshared and the emails also may have helped to raise general awareness of the existence of *CSD Communications*.

**Figure 10:**
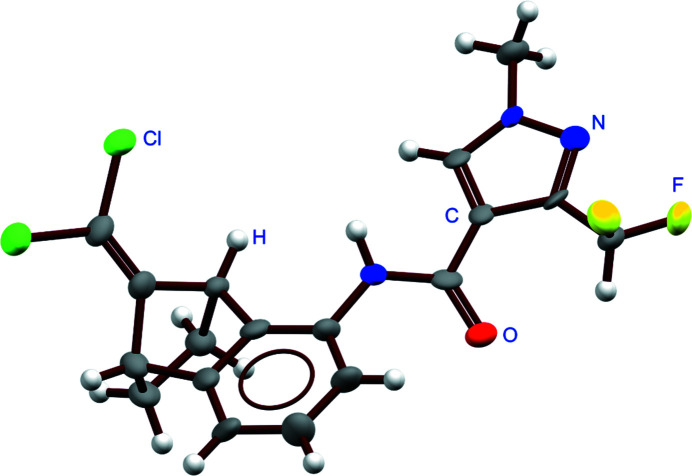
*ORTEP*-style *Mercury* rendering of CSD-UZIJUK.

The growth in the number of crystal structures shared as *CSD Communications* has also led to an increase in the diversity of authors of these types of datasets, as more people learn about *CSD Communications* and appreciate the benefits. Although the majority of *CSD Communications* originate from academic institutions, the collection now includes structures that originated from industry. This is particularly significant since the proportion of industrial data in the entire CSD is very low (estimated to be <1%) and suggests that *CSD Communications* may provide a slightly easier route for industry to share their data. The low percentage of industrial data in the CSD may reflect the proportion of crystallographers in industry compared with academia and the sensitivity of publishing work arising from industry due to intellectual property considerations. Some examples of *CSD Communications* published by industry include CSD-MATXUD, a structure of tesaglitazar shared by Astra Zeneca that has been used in clinical trials (Black & Pettersen, 2017 ▸); CSD-UZIJUK, a structure of benzovindiflupyr shared by Syngenta that is used as an agricultural fungicide (Fig. 10 ▸) (Keates, 2016 ▸); and CSD-JAYSAG, a structure of 3,4,5-tris­(benzyl­oxy)-2-(ethyl­sulfanyl)-6-methyl­oxane shared by Pfizer (Back & Doherty, 2017 ▸).

In a bid to increase the volume and diversity of the collection, a further initiative has been established at the CCDC that enables crystallographers to deposit historic data in hard-copy format with the CCDC undertaking the work to convert the data into CSD entries. This work is especially valuable in ensuring that legacy structures (in some cases resulting from the retirement of crystallographers), which were not published or available in electronic format previously, can be made available to the community.

Although the popularity of both *CSD Communications* as a format to publish crystallographic data and the list of authors who are taking advantage of this option is growing, most of the data published in this way still come from only a few prolific depositors. So, the question stands: if the few can deposit in such magnitude, then how many more datasets are still out there waiting to be published?

## 5. Challenges

### 5.1. Avoiding mis-publications

*CSD Communications* provide a method of sharing crystallographic data that is unlikely to go on to be published in a scientific article. However, a proportion of *CSD Communications* do end up also published in the literature. There could be many reasons for this including researchers misunderstanding the process or simply subsequent decisions that mean the data are included in an associated article. To try to address any misunderstanding, a confirmation box has been added to the deposit page to clarify the role of *CSD Communications* and an additional email is sent to the depositor to alert them when their dataset is made publicly available in this way.

### 5.2. Enhancing additional information about a structure

As described earlier, entries in the CSD are often enhanced with additional information. This can be provided by the authors during deposition or may be identified by the CSD editors during curation of the structure after publication. Due to the nature of *CSD Communications*, the only opportunity to obtain such information is from the authors during deposition. One example of additional information is bioactivity and natural source data. Fewer *CSD Communications* have bioactivity and natural source information than in the CSD as a whole. This could be due to a trend in the type of compounds or chemicals that become *CSD Communications* or to do with a lack of accompanying publications where this additional information could be found. This additional information can be useful when data-mining the structures and it is therefore encouraged for depositors to add as much information about a structure as possible.

### 5.3. Setting the right criteria for *CSD Communications*

Currently there are no additional criteria for depositing *CSD Communications*. If the data meet the remit of the CSD and are contained in CIF format, authors can publish their data as a *CSD Communication*. However, with increasing incentives for publishing data this could also increase the potential for fraudulent datasets so it will be important for the CCDC to consider what criteria to set in the future for this collection. With most refinement software now including *hkl* and *res* data in the CIF by default, it may be that mandating this information could be considered in the future to help identify issues with data integrity. It may also be considered if additional metadata should be mandatory for these structures to further enhance the FAIRness (Wilkinson *et al.*, 2016 ▸) of the collection. These considerations will need to balance the drive to lower the barriers to data sharing while increasing the integrity of the collection.

### 5.4. Increasing data sharing

There are a number of challenges to overcome to enable researchers to share more data and it is important to reflect on what the barriers might be in order to consider how these could be surmounted in the future.

The first barrier is often down to ownership of the data, which can be complex. Many service crystallographers collect and refine data on behalf of another primary investigator, and although they may feel able to deposit the data, they do not feel that they hold the permission to make the data public. It is therefore key to ensure that standard recommendations exist for sharing crystallographic data and that these are communicated more widely than just within the crystallographic community. This could, for example, help to set expectations that if the data have not been shared after a 4 year ‘in-house’ embargo, they are automatically shared through the appropriate database unless the primary investigator proactively extends the embargo in yearly increments. With expectations in place for the publication of crystal structures via *CSD Communications*, both the primary investigator and the crystallographer will be in a better position to retain control of their data and to determine the stage at which the data should be made public. This should also encourage best practice within the community and avoid the automatic addition of unfinished or low-quality datasets after a set number of years.

Another barrier to data sharing is often associated with data quality. For some datasets, the primary purpose of the structure is to confirm the chemical connectivity of the solid form. Once the chemistry has been confirmed then the dataset may be deemed good enough for its purpose but not deemed of high enough quality to share more widely. The crystallographer may not have time to improve the quality of the refinement if they do not intend to publish in an associated article or, in some cases, the structure may be particularly difficult or challenging to solve and the data quality is already as good as possible under the circumstances. In these cases, it is important that there are still routes available to share the data. This can be of the structural model or the raw experimental data. While *CSD Communications* are a mechanism to share an available structural model, the new ‘Raw Data Letters’ section in the *IUCrData* journal allows the sharing of raw experimental data with interesting features, while giving a citable attribution to the authors (Kroon-Batenburg *et al.*, 2022 ▸). In terms of the CSD, consideration needs to be given to sharing such crystal structures without impacting the overall value of the collection. For example, future consideration should be given to whether some structures should be shared through basic look-up tools but not go on to contribute to knowledge bases derived from data in the CSD. This could lead to additional filters and flags being applied to the CSD to enable users to select appropriate data for their research needs.

## 6. Conclusions

In recent years, the *CSD Communications* publication mechanism has grown to be a significant dissemination channel for crystal structure data. Sharing data via this route provides authors with the opportunity to publish and be credited for valuable, otherwise orphaned data, while simultaneously benefitting the community by providing access to additional crystallographic information. Here we have demonstrated that the structures represented by *CSD Communications* encompass the full range of chemistry covered by the CSD and are deposited from all over the world; however, two thirds of *CSD Communications* contain substances not otherwise contained in the full CSD. Thus, an initiative like *CSD Communications* can open a new pathway for data sharing and has contributed to both the breadth and the depth of the CSD. The large increase in the number of *CSD Communications* over the last 5 years has proved that there is a significant amount of data that would otherwise remain unshared through standard publishing routes. It has been shown that the quality of data contained in *CSD Communications* is typically in line with CSD entries sourced from peer-reviewed journals, which supports the reliability of these structures for reuse in further research. Depositors are encouraged to help maintain this quality by submitting completed and validated entries, including full author lists, enhanced information about the compound and to quote previous deposition numbers on revisions. Alternatively, structures can easily be shared as *CSD Communications* via updating ‘My Structures’. The publication of *CSD Communications* is rapid, free and the data are immediately publicly available on direct deposition via this route. The broader scientific community is encouraged routinely to cite *CSD Communications*, when appropriate, in their peer-reviewed works. In order to facilitate referencing and discoverability, *CSD Communications* adhere to archival standards, have an ISSN number and each entry includes a DOI, together with the author list and crystallographer details as supplied by the depositor. Studies on the CSD that have relied on these data [*e.g.* a recent paper on thermal expansion (van der Lee & Dumitrescu, 2021 ▸)] show the value of this growing collection and how sharing these datasets can help to advance science for public benefit. However, we believe that the current number of structures available through *CSD Communications* is still a small proportion of the wealth of datasets that remain unpublished. This review stands as a call to action for all crystallographers to take the time to share more data as their gift to science so that new insights and discoveries are possible, and collectively we can work together to advance science.

## Supplementary Material

Additional figures and information to support the statistics in the paper. DOI: 10.1107/S2052252522010545/lt5055sup1.pdf
