# Supplementary material for: CSD Communications of the Cambridge Structural Database
Source: IUCrJ. 2023 Jan 1;10(Pt 1):6–15. doi: 10.1107/S2052252522010545 (PMC9812213; doi:10.1107/S2052252522010545)
Supplement: Supplementary file 1 [file m-10-00006-sup1.pdf]

IUCrJ

Volume 10 (2023)

Supporting information for article:

CSD Communications of the Cambridge Structural Database

Gregory M. Ferrence, Clare A. Tovee, Stephen J.W. Holgate, Natalie T. Johnson, Matthew P. Lightfoot, Kamila L. Nowakowska-Orzechowska and Suzanna C. Ward

S1. Additional data

Figure S1: Change since 1990 in the percentage of new CSD Communications deposited each year that are organic or metal-organic.

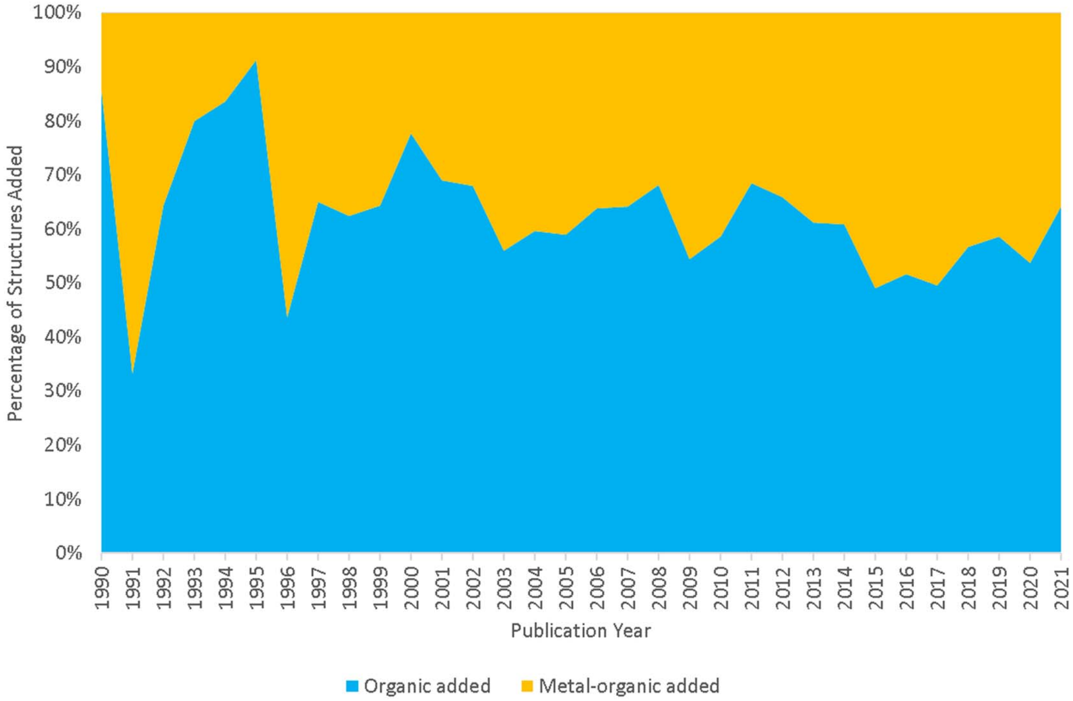

Figure S2: illustrates that the average R-factor value is not constant and fluctuates for *CSD Communications*, whereas the value for the whole CSD is more constant as a greater number of structures lessen the effects of any outliers. Plot showing the change in R-factor since 1990 for CSD Communications (blue) and the CSD (dark grey)

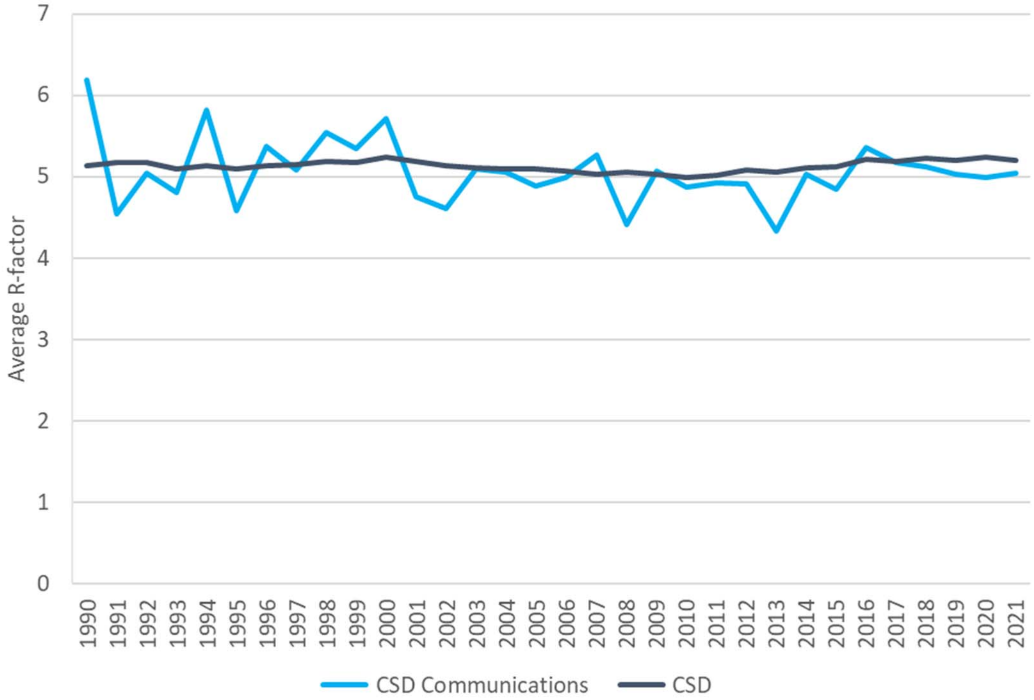

Table S1: Table with the number of structures in the CSD Subsets that are CSD Communications or in the whole CSD (June 2022, v5.43 CSD). Totals: 46616 CSD Comms, Total CSD: 1161919

**CSD Subset**	CSD Communications	(%)	Full CSD	(%)
**Teaching Subset**	17	0.04%	837	*0.07%*
**Drug**	511	1.10%	13791	*1.19%*
**Single component drug**	105	0.23%	2239	*0.19%*
**MOF**	3244	6.96%	114373	*9.84%*
**Non-disordered MOF**	2470	5.30%	86231	*7.42%*
1d	986	2.12%	33152	*2.85%*
2d	681	1.46%	24206	*2.08%*
3d mof	787	1.69%	27463	*2.36%*
**Pesticides**	57	0.12%	970	*0.08%*
**COVID19**	8	0.02%	267	*0.02%*
**Best R factor**[a]	28717	61.60%	677043	*58.27%*
**Best hydrogens**[a]	28173	60.44%	677043	*58.27%*
**Best low temp**[a]	28801	61.78%	677043	*58.27%*
**Best room temp**[a]	27650	59.31%	677043	*58.27%*
Polymorph	1528	3.28%	38377	*3.30%*
Electron	8	0.02%	168	*0.01%*
High pressure	58	0.12%	3777	*0.33%*
hydrate	4218	9.05%	146638	*12.62%*
Adps available	41752	89.57%	865927	*74.53%*

[a] Best representative subsets from van de Streek, 2006.

S2. Refcode Families

29,084 refcode families only contain CSD Communications (out of 44,678 refcode families for CSD Comms, 65% of families only contain CSD comms).

Total number of CSD Communications that are in those refcode families that only contain CSD communications: 29,746 (out of 46,616 CSD Communication entries, 64%).
